# Increased cytoplasmatic expression of cancer immune surveillance receptor CD1d in anaplastic thyroid carcinomas

**DOI:** 10.1002/cam4.2573

**Published:** 2019-09-27

**Authors:** Florian Weber, Henrik Junger, Jens M. Werner, Natalia Velez Char, Carolina Rejas, Hans J. Schlitt, Matthias Hornung

**Affiliations:** ^1^ Department of Pathology University Hospital Regensburg Regensburg Germany; ^2^ Department of Surgery University Hospital Regensburg Regensburg Germany

**Keywords:** CD1d, immunotherapy, lymphocyte infiltration, NKT cell, thyroid carcinoma

## Abstract

**Background:**

Anaplastic thyroid carcinomas are associated with rapid tumor growth, short survival time and without any promising therapy to improve the poor prognosis. In this study, expression of immunoregulative receptor CD1d and lymphocyte infiltration in different thyroid tumors as well as in healthy tissue were analyzed in order to find new targets for an immunotherapeutic approach.

**Methods:**

CD1d immunohistochemistry was performed in samples of 18 anaplastic, 17 follicular, 27 papillary, and 4 medullary thyroid carcinomas as well as in 19 specimens from normal thyroid tissue and additionally in 10 samples of sarcoma, seven malignant melanoma and three spindle‐cell lung carcinoma. Furthermore, thyroid samples were stained with antibodies against CD3, CD20, CD56, CD68, and LCA in order to analyze lymphocyte infiltration.

**Results:**

For the first time CD1d receptor expression on normal thyroid tissue could be demonstrated. Moreover, anaplastic thyroid carcinomas showed significantly higher expression levels compared to other thyroid samples. Most astonishingly, CD1d expression disappeared from the cellular surface and was detected rather in the cytoplasm of anaplastic thyroid carcinoma cells. In addition, histologically similar tumors to anaplastic carcinoma like sarcoma and malignant melanoma revealed distinct CD1d staining patterns. Furthermore, infiltration of T cells, B cells, and macrophages in anaplastic thyroid carcinomas was different when compared to normal thyroid tissue and all other thyroid carcinomas.

**Conclusions:**

Anaplastic thyroid carcinomas show significantly higher expression of CD1d, a receptor for NKT cells, which are subject of several anticancer therapy studies. These results may offer a novel approach to explore immunotherapeutic treatment options.

## Graphical abstract

Anaplastic thyroid carcinomas show significantly higher expression of CD1d, a receptor for NKT cells, which are subject of several anticancer therapy studies. For anaplastic thyroid carcinomas, this may offer a new differentiation marker as well as a novel approach to explore immunotherapeutic treatment options.

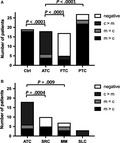

## 1 INTRODUCTION

The incidence of malignant thyroid tumors has increased over the last few years.^1^ These tumors represent a very heterogeneous group of malignancies, with distinct differences in histology and likewise in clinical outcome.^1^, ^2^ Most thyroid malignancies are described as follicular or papillary thyroid carcinomas (FTC/PTC) but about 1.7% of the patients are diagnosed with anaplastic thyroid carcinoma (ATC).^3^ ATC is a rare, yet rapidly growing neoplasm of the thyroid gland with a poor prognosis.^4^ In contrast to FTC or PTC, which can be treated postoperatively by radioiodine therapy, ATCs are not sensitive to any further treatment and therefore have the worst prognosis with a median survival of only 5 months.^3^, ^5^ However, immunotherapy as new therapeutic approach has been introduced against a wide range of solid tumors recently. Oncologic trials have focused on developing immunomodulating therapies to restore the functional ability of different immune cells against neoplastic cells and showed promising results in several tumor types, such as lung cancer, melanoma, and colon cancer.^6^, ^7^ Regarding malignant thyroid tumors, recent advances approached several strategies for immunomodulation, such as inhibiting recruitment of tumor‐associated macrophages (TAM), blocking and inhibiting of several immune checkpoints, as well as identification of tumor‐specific antigens.^8^

In this context, natural killer T (NKT) cells, which are considered to be regulators of an inflammatory immune response, also represent a promising target for antitumor therapy.^9^, ^10^, ^11^ They are a subset of lymphocytes that co‐express NK cell markers and the T‐cell receptor.^12^ In humans, NKT cell population expresses the Vα24 and Jα18 gene segments, which are preferentially associated with Vβ11.^13^, ^14^ NKT cells are activated through a specific receptor called CD1d, which belongs to a group of CD1 molecules associated with β2‐microglobulin, and is known to present lipids, including glucosylceramides and glycosylphosphatidylinositol to NKT cells.^15^ It is already known that activation of CD1d leads to the production of interleukin (IL)‐4 and interferon (IFN)‐g and an increase in the cytolytic activity of NKT cells.^16^ However, expression of CD1d, a MHC class I‐related molecule, could just be shown on hematopoietic cells and on epithelial cells of the intestine.^9^, ^17^, ^18^

In this study, the expression of CD1d and lymphocyte infiltration in healthy thyroid tissue as well as in several thyroid carcinomas was analyzed in order to find new targets for an immunotherapy approach.

## 2 MATERIALS AND METHODS

### 2.1 Tissue samples

FFPE tissue samples were collected from the archives of the Institute for Pathology, Regensburg. Tumor entity was confirmed before anonymization. Two tissue micro arrays (TMAs), containing a total of 18 samples of ATC, 17 samples of FTC (12 grossly invasive, 5 minimally invasive), 27 samples of PTC, and 4 samples of medullary thyroid carcinoma (MTC), were created with subsequent histomorphological control of the tumor samples by a trained pathologist (FW) using conventional H&E staining. The TMAs also contained 19 samples of normal thyroid tissue, as well as 10 samples of sarcoma (SRC; two myofibroblastic sarcomas, two dedifferentiated liposarcomas, and one fibrosarcoma, leimyosarcoma, biphasic synovial sarcoma, clear cell sarcoma, carcinosarcoma and sarcomatoid renal cell carcinoma, respectively), seven samples of malignant melanoma (MM), and three samples of spindle‐cell lung carcinoma (SLC).

### 2.2 Immunohistochemistry

**Figure 1:**
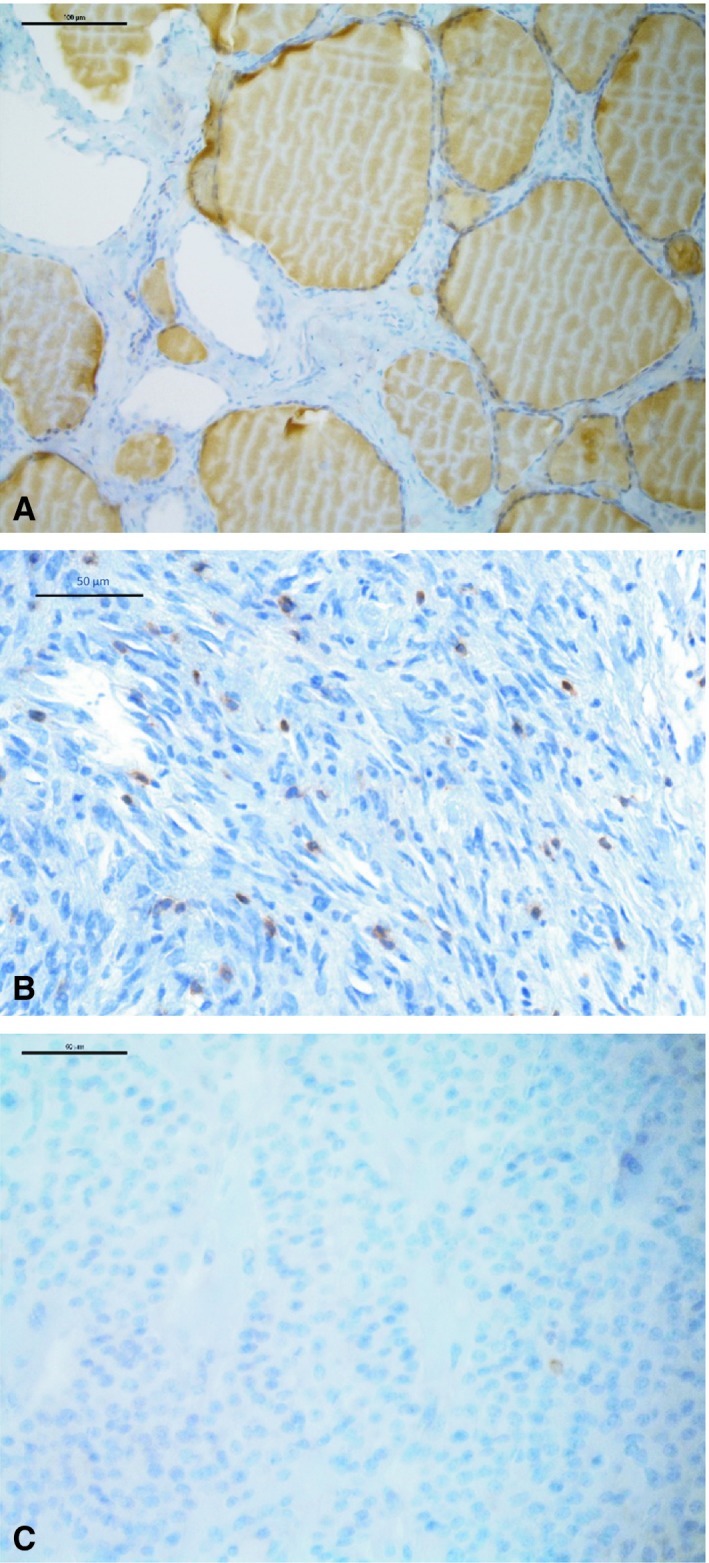
Examples of CD3 immunohistochemistry are shown for normal thyroid tissue (A), ATC with numerous positive lymphocytes (B) and FTC with only one positive lymphocyte (C)

For standard immunohistochemistry, 5 µm thick paraffin sections were processed using routine diagnostic antibodies and immunostaining protocols (Ventana immunostainer) of the Institute for Pathology, Regensburg for staining of CD3, CD20, CD56, CD68 (KP1), and LCA (CD45). The number of positively stained cells (lymphocytes or monocytes) per high‐power field (HPF, 400x magnification) for each tissue sample was recorded and used for further analysis. For CD1d immunohistochemistry, a mouse monoclonal antibody against human CD1d (LifeSpan BioSciences, code: LS‐B6766, clone: ms R3) was used in a dilution of 1:50. The dewaxed paraffin sections were subjected to heat‐induced epitope retrieval for 5 minutes at 120°C in Tris‐EDTA buffer at pH 8.5. The antigen localization was carried out using polymer‐based detection systems and an automated system setting with diaminobenzidine as the final substrate. Immunohistochemistry was evaluated by an expert pathologist (FW); for CD3, CD20, CD56, CD68, and LCA, the number of specifically positive cells per high‐power field (HPF, 400×) were counted. For CD1d, in addition to the number of specifically positive cells per HPF, the immunostaining of tumor/normal thyroid cells was assessed by an expert pathologist (FW) and a trained pathologist (NVC) according to relative intensity of cytoplasmic (c) and membranous (m) staining, that is c > m, c = m, or m > c; some samples showed a completely negative staining reaction, and only one sample had nuclear positivity (Figure 1). In case of differing results between the two observers, a mutual consensus was found and recorded.

### 2.3 Microscopy

Stained sections were viewed with a Leica light microscope (Leitz DMRBE; Leitz). Images were acquired using a MIRAX digital slide scanner (Zeiss).

### 2.4 In situ hybridization

mRNA detection of CD1d (Hs‐CD1D #496771, ACDBio) was performed using the RNAscope 2.5 HD RED Reagent Kit (#322350, ACDBio) on 3‐μm FFPE sections of tissue micro arrays (TMAs) according to the manufacturer's instructions. RNA quality was evaluated for each sample with a probe specific to the housekeeping gene ubiquitin (Hs‐UBC, #310041, ACDBio). Negative control background staining was evaluated using a probe specific to the bacterial DapB gene (DapB, #310043, ACDBio). Only samples with an average of >4 dots per cell with the housekeeping gene probe staining and an average of <1 dot per 10 cells with the negative control staining were assayed with target probes. Analysis was performed by two independent researchers on a Leica light microscope (Leitz DMRBE; Leitz) using a 40× objective. The RNAscope signal is scored on the basis of number of dots per cell as recommended by the manufacture (ACDscore) and was applied as follows 0 = 0 dot/cell, 1 = 1‐3 dots/cell, 2 = 4‐9 dots/cell, 3 = 10‐15 dots/cell, and 4 = >15 dots/cell with > 10% of dots in clusters).

### 2.5 Statistical analysis

D'Agostino & Pearson normality test did not confirm a normal distribution of the data set. Therefore, results are shown as median ± interquartile range (IQR). Statistical analyses were performed using a nonparametric Kruskal‐Wallis test followed by Dunn's multiple comparison. Differences were considered significant at *P* < .05. GraphPad Prism 7.0b (GraphPad Software, Inc) was used for significance tests and generating plots.

### 2.6 Ethics

The study had been approved by the institutional review board of the University Hospital of Regensburg (# 14‐160‐0029).

## 3 RESULTS

### 3.1 Immune cell populations in normal thyroid tissue

Initially, normal thyroid tissue was stained for several immune cell populations by immunohistochemistry. As shown in Figure 1, using specific antibodies for different immune cell markers normal thyroid tissue showed a low number of T cells (CD3 pos., 3 ± 4.1 per HPF), macrophages (CD68 pos., 2.06 ± 1.8 per HPF), and B cells (CD20 pos., 0.33 ± 0.8 per HPF).

### 3.2 Lymphocyte infiltration and thyroid cancer

**Figure 2:**
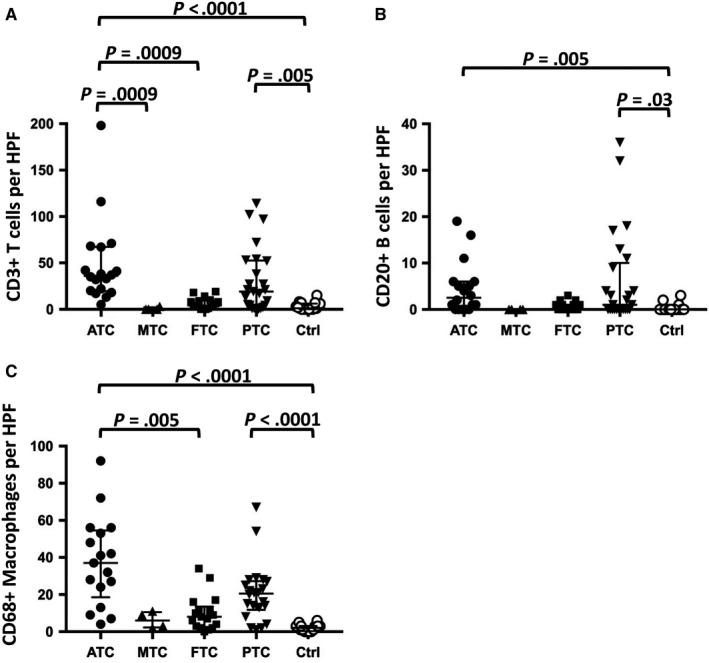
Mean number of positive cells per HPF for CD3 (A), CD20 (B) and CD68 (C) in ATC, FTC, MTC, PTC, and normal thyroid tissue

Then, different thyroid carcinomas were stained for cells of the immune system (Figure 1). As summarized in Figure 2, ATC specimens showed a significantly higher number of CD3 (36, IQR 19.5‐67.25) and CD68 (37, 18.5‐54.5)‐positive cells in comparison to FTCs (CD3: 4; 2‐9.5, *P* = .0009; CD68: 1, 0‐3, *P* = .005), PTCs (CD3: 19, 3‐52.5, n.s.; CD68: 20.5, 11.75‐27.25, n.s.) and MTCs (CD3: 0, 0‐2.25, *P* = .0009; CD68: 6, 2.25‐10.5, n.s.), as well as normal thyroid tissue (CD3: *P* < .0001; CD68: *P* < .0001). In addition, cells expressing CD20, a B cell marker, could be detected significantly more frequently in ATCs (2.5, 0‐6) than in normal thyroid tissue (*P* = .005) but the differences to FTCs, PTCs, and MTCs were not significant. Interestingly, FTCs revealed similar numbers of CD3‐positive cells when compared to normal thyroid tissue, but less than PTCs (Figure 2).

### 3.3 CD1d expression on thyroid tissue

**Figure 3:**
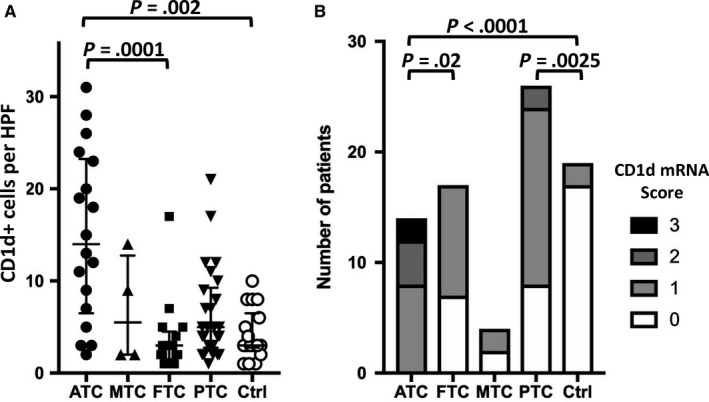
A, Mean number of positive immune cells per HPF for CD1d and (B) CD1d mRNA (Score 0‐3) in ATC, FTC, MTC, PTC, and normal thyroid tissue

**Figure 4:**
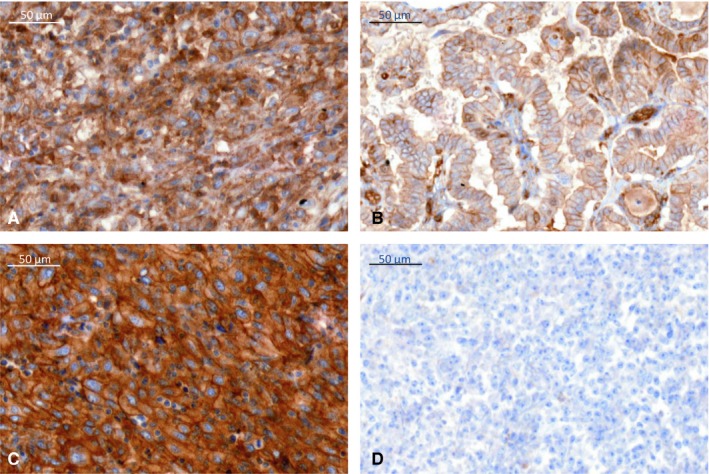
Different staining patterns for CD1d immunohistochemistry with predominantly cytoplasmic staining in ATC (A), predominantly membranous staining in PTC (B), equally membranous and cytoplasmic staining in SRC (C) or no staining in FTC (D)

NKT cells could not be detected in thyroid tissue, most likely due to the fact that to our knowledge it has not yet been possible to stain these specific cells by immunohistochemistry. Nevertheless, the expression of the NKT cell activating receptor CD1d.^19^, ^20^ was analyzed and as expected, there were more immune cells expressing CD1d in ATCs (Figure 3). Furthermore, for the first time CD1d could be detected in thyroid tissue. Staining of normal thyroid tissue revealed membranous expression of CD1d (Figure 4) as it has been already shown for hematopoiesis‐derived and intestinal epithelial cells.^17^, ^18^, ^21^

### 3.4 CD1d expression and thyroid cancer

**Figure 5:**
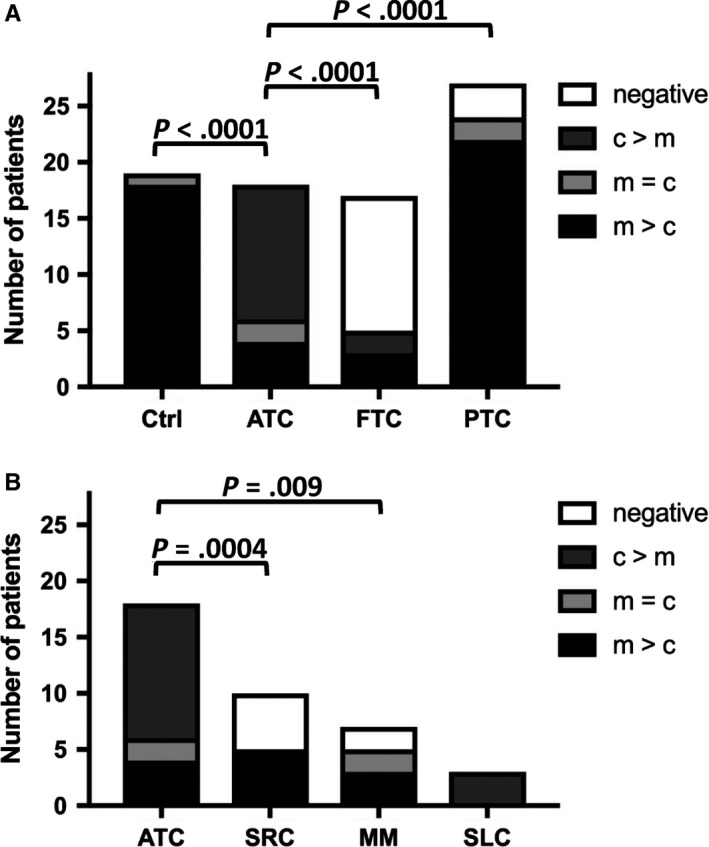
Staining patterns of normal thyroid tissue and different thyroid cancer types (ATC: anaplastic thyroid carcinoma; FTC: follicular thyroid carcinoma; MTC: medullary thyroid carcinoma; PTC: papillary thyroid carcinoma) (A) as well as other malignant tumors (SRC: sarcoma; MM: malignant melanoma; SLC: spindle‐cell lung cancer) (B). Staining patterns are indicated as predominantly membranous (m > c), predominantly cytoplasmic (c > m), equally membranous and cytoplasmic (c = m), negative (neg.) and nuclear (n)

Additionally, different thyroid carcinomas were stained for CD1d expression (Figure 4). Interestingly, ATC was associated with a significantly higher number of CD1d‐positive cells compared to other tumor entities and normal thyroid tissue. Additionally, looking at the respective staining pattern for CD1d in individual tumor cells, different characteristic features with regard to the localization of CD1d expression either in the cytoplasm or on the cell membrane could be found (Figure 5A). In the control group of 17 normal thyroid tissue samples, all showed a membranous staining pattern for CD1d expression (m > c 17/17). However, ATC cells showed a very strong and predominantly cytoplasmatic staining of CD1d expression (12/18 cases with c > m, 4/18 with m > c, 2 with c = m). FTC cells stained almost completely negative (12/17 neg.), with only five cases of either predominantly membranous (3/17) or cytoplasmic staining (2/17). MTC cells were mostly stained homogeneous membranous and cytoplasmic (3/4 with c = m), only one case was negative for CD1d. PTC cells showed a similar staining as normal thyroid tissue with a predominantly membranous staining (22/27 with m = c) and even partial nuclear staining (1/27), as well as two completely negative stainings (2/27).

### 3.5 CD1d mRNA expression in various thyroid cancer types

**Figure 6:**
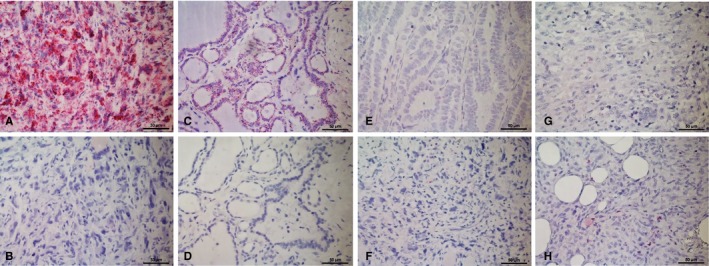
Expression of UBC (positive control) and DapB (negative control) with red chromogenic ISH (RNAscope) in a representative ATC (A and B) and normal thyroid tissue (C and D). Different levels of CD1d mRNA detected with red chromogenic ISH (RNAscope) in PTC (ACD mRNA score 1) (E) and several ATC (ACD mRNA score 1, 2 and 3) (F, G and H). All images at 4× magnification

CD1d transcripts were detected by chromogenic alkaline phosphates‐based in situ hybridization in FFPE tissue (RNAscope). All study section showed very good mRNA quality, indicated by very high abundant signal (mRNA score >3) for the positive control ubiquitin (Figure 6A), and further high assay quality was revealed by no unspecific signal (mRNA score = 0) for the negative control DapB (Figure 6B). Figure 6 shows representative areas of CD1d mRNA expression in ATC, FTC, MTC, PTC, and normal thyroid tissue. CD1d was almost exclusively expressed, at relatively high levels, in ATC; the lowest CD1d mRNA expression was seen in normal thyroid tissue (Figure 3B), consistent with our IHC data (Figure 4). CD1d mRNA expression in ATC was significantly higher than that in FTC (*P* = .02) and normal thyroid tissue (*P* < .0001; Figure 3B). FTC and MTC showed only sporadic CD1d mRNA expression levels (Figure 3B). In contrast to FTC and MTC, PTC showed a moderately increased CD1d mRNA expression, significantly higher than that in normal thyroid tissue (*P* = .0025; Figure 3B).

### 3.6 CD1d expression as a diagnostic tool

**Figure 7:**
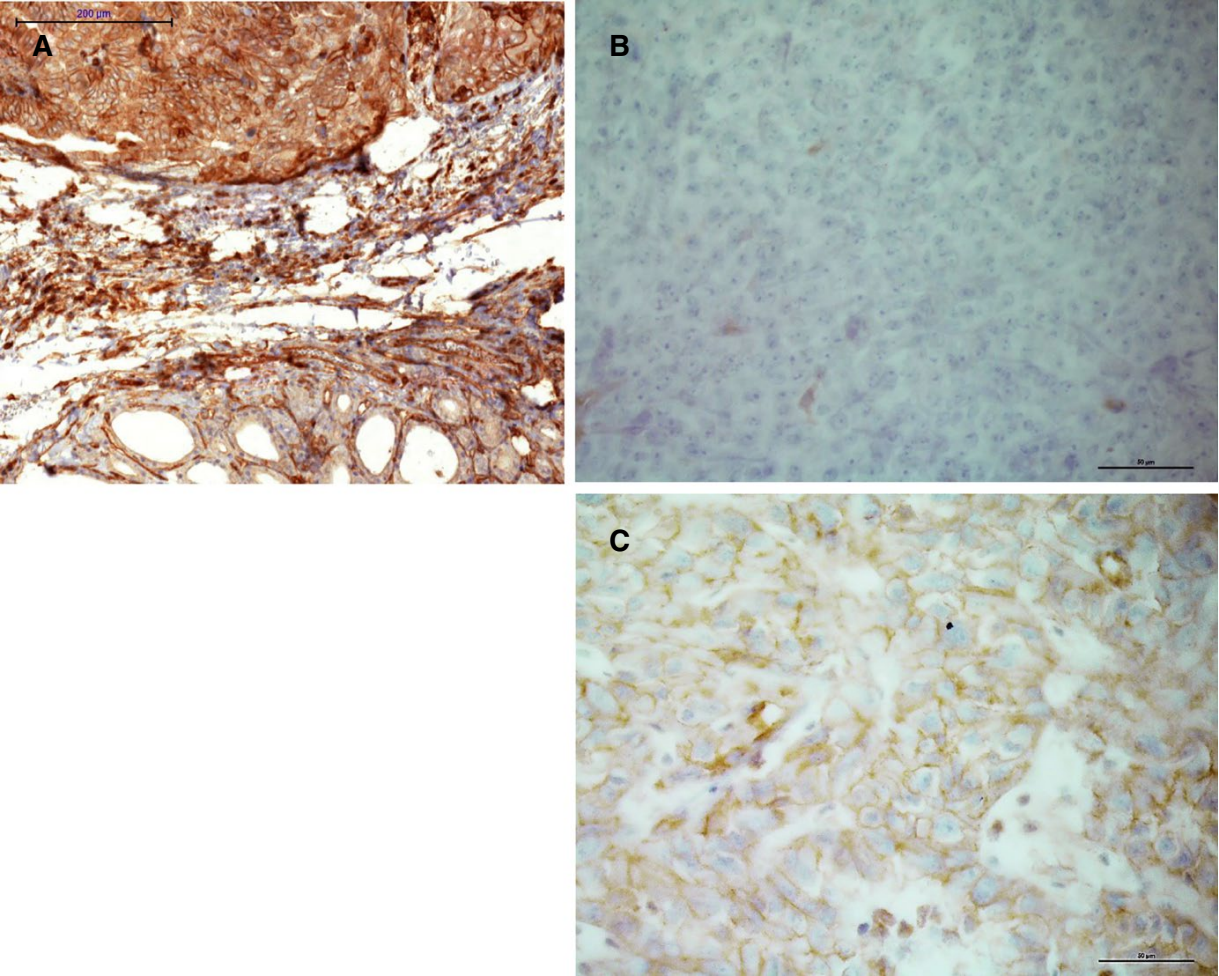
CD1d‐positive staining in ATC (A) compared to negative staining in MM (B) and weak membranous staining in SRC (C)

Since CD1d expression was very strong and localized predominantly cytoplasmic in ATC cells, we next wanted to assess if this could be used as histopathological tool to help in the differential diagnosis of ATC and other mediastinal tumors. Therefore, specimens of sarcoma (SRC), MM, and SLC were examined for their respective staining profiles (Figure 7). SRC cells were either negative (6/11) or showed a predominantly membranous staining pattern (5/11), while MM cells showed a heterogeneous profile with all patterns present (3/7 with m > c, 2/7 with c > m, 2/7 neg.). All three SLC cases showed a predominantly cytoplasmic staining pattern (3/3) (Figures 5B and 6).

## 4 DISCUSSION

In this study, for the first time CD1d receptor expression could be detected on thyroid tissue. So far, expression of CD1d was described only on hematopoietic and epithelial cells of the intestine.^9^, ^17^, ^18^ Furthermore, analysis of different thyroid tumor entities revealed significant higher expression of CD1d and CD1d mRNA in ATCs compared to normal thyroid tissue and all other thyroid tumor types. On average, patients with ATCs survive only a few months after primary diagnosis.^22^ Although radiotherapy and different chemotherapies result in slightly improved survival rates,^23^, ^24^ outcome is still very poor.^22^ Therefore, several treatment strategies are currently under research.^22^ Regarding cancer therapy, in the last few years a lot of effort was put into the research of immunotherapy with NKT cells, for which CD1d serves as target receptor.^11^, ^25^, ^26^, ^27^ Activation pathways of NKT cells in cancer, CD1d mechanism, and even CD1d escape strategies in tumors are topics of current studies.^28^ In this regard, a CD1d‐binding glycolipid for specific stimulation of iNKT cells has been developed for the treatment of breast cancer.^29^ Interestingly, CD1d expression in ATCs was not located on the cell surface but rather in the cytoplasm of the tumor cell. It has already been shown that CD1d expression is decreased in several diseases. Virus infection inhibits NKT cell‐CD1d function among others through downregulation of CD1d.^30^, ^31^ In this context, herpes simplex virus 1 phosphorylates the type II kinesin motor protein KIF3A which lead to the suppression of CD1d.^32^ Otherwise, in systemic lupus erythematosus TLR9‐induced microRNA‐155 and Ets‐1 decrease membrane CD1d levels.^33^ But also in malignancies CD1d degradation could be observed. In chronic myeloid leukemia, activation of the Rho‐associated protein kinase (ROCK) mediates the reduction of membrane expression of CD1d but not the reduction of intracytoplasmatic levels or mRNA transcripts^34^ and a certain kind of invasive breast cancer is also associated with downregulated CD1d.^35^ Regarding the CD1d receptor molecule, a Thr/Ser residue motif in the cytoplasmatic tail seems to be pivotal for the surface expression.^36^ Nevertheless, future research has to focus on the mechanism of CD1d downregulation, especially in ATCs. Although CD1d downregulation seems to represent dedifferentiation, a correlation between cytoplasmatic, respectively membranous expression and survival time could not be found; this is may be due to the limited number of patients.

Infiltrating lymphocytes and their function related to different pathologies are subject to ongoing research. Concerning the thyroid gland, lymphocytes are mainly analyzed in Grave's disease and Hashimoto's thyroiditis due to the autoimmune background.^37^ These data were compared to lymphocyte infiltration into thyroidal malignant tumors, mainly focused on the immunoregulatory potential.^37^, ^38^, ^39^ Although previously published data revealed no increase of lymphocytes in ATCs compared to other thyroid carcinomas,^40^, ^41^ Lindhorst et al^42^ showed clear evidence that lymphocyte infiltration is more frequent in ATCs, which can be confirmed by results of the present study. Interestingly, in ATC patients, myeloid‐derived suppressor cells are increased in the peripheral blood and cell‐mediated immune responses seem to be suppressed.^43^ Furthermore, in ATCs, increased secretion of CXCL10 has been reported.^44^ In turn, CXCL10 binds to CXCR3 subsequently leading to T and NKT cell activation.^45^, ^46^ In addition to its function in coordinating inflammation and immune response, it has been already shown that CXCR3 and its ligand CXCL10 are involved in the progression of several different cancer types.^47^, ^48^, ^49^, ^50^, ^51^, ^52^ However, further research will be required to evaluate if immunotherapy can be an option for ATCs in the future and to prove if CD1d represents an appropriate target for such treatment.

An interesting finding in our study is the possibility to use CD1d immunohistochemistry in an extended panel for the differential diagnosis of mediastinal lesions in a difficult clinicopathologic setting. Due to its location near the upper mediastinum and often advanced stage with infiltration of other organs and structures in the vicinity, it can be difficult to distinguish ATC from other malignant neoplasms in the mediastinal area or metastases, such as poorly differentiated squamous carcinoma (SCC) of the head and neck,^53^ sarcoma (SRC),^54^, ^55^ malignant melanoma (MM),^56^ sarcomatoid mesothelioma or lung cancer.^57^ In some cases, diagnosis will be possible upon histopathologic examination alone, while other cases require additional immunohistochemical analysis. Aside from poorly differentiated SCC with positive epithelial markers, the distinction between SRC, MM, and ATC can be a problem when other specific markers, for example HMB45 or Melan A, are also negative. Additionally, vimentin has been reported to be positive in a number of ATC.^58^, ^59^ A strong, predominantly cytoplasmic positivity for CD1d seems very characteristic for ATC, while other poorly differentiated neoplasms that can occur in the mediastinum, for example undifferentiated sarcomas or metastases of malignant melanoma, show either negative staining for CD1d or a predominantly membranous pattern. We therefore suggest adding the immunohistochemical staining for CD1d to a wider panel of different cytokeratins, melanocytic markers, thyroid markers (such as TTF‐1 and PAX8) and lymphocytic antigens to help establishing the diagnosis of ATC. Further, our findings should be confirmed in immunohistochemical studies with larger numbers of ATC.^60^

The scope of our study is certainly limited by the small amount of cases that were included. For further investigations regarding the significance of different levels and locations of CD1d expression, a larger number of ATC cases needs to be surveyed. Also, in this study the correlation between CD1d expression on different thyroid malignancies and its possible prognostic and therapeutic impact was not investigated. This should be addressed in further studies.

## CONFLICT OF INTEREST

Florian Weber, Henrik Junger, Jens M. Werner, Natalia Velez Char, Carolina Rejas, Hans J. Schlitt, and Matthias Hornung have no conflict of interest to disclose.

## AUTHOR CONTRIBUTIONS

FW and HJ contributed eaqually to this work. FW, HJ, MH, HS, and JW conceptualized and designed the study; involved in analysis and interpretation of the data, and drafting of the manuscript; revised the manuscript critically. FW, HJ,  MH, and JW performed data acquisition and drafted the manuscript. NVC and CR performed experiments. All the authors had access to the study data and critically reviewed and approved the final version of the manuscript.

## Data Availability

The data that support the findings of this study are available from the corresponding author upon reasonable request.
